# Pathogen invasion history elucidates contemporary host pathogen dynamics

**DOI:** 10.1371/journal.pone.0219981

**Published:** 2019-09-19

**Authors:** Vance T. Vredenburg, Samuel V. G. McNally, Hasan Sulaeman, Helen M. Butler, Tiffany Yap, Michelle S. Koo, Dirk S. Schmeller, Celeste Dodge, Tina Cheng, Gordon Lau, Cheryl J. Briggs

**Affiliations:** 1 Department of Biology, San Francisco State University, San Francisco, California, United States of America; 2 Museum of Vertebrate Zoology, University of California Berkeley, Berkeley, California, United States of America; 3 Center for Biological Diversity, Oakland, California, United States of America; 4 EcoLab, Université de Toulouse, Toulouse, France; 5 Department of Ecology Evolution and Marine Biology, University of California Santa Barbara, Santa Barbara, California, United States of America; University of South Dakota, UNITED STATES

## Abstract

Amphibians, the most threatened group of vertebrates, are seen as indicators of the sixth mass extinction on earth. Thousands of species are threatened with extinction and many have been affected by an emerging infectious disease, chytridiomycosis, caused by the fungal pathogen, *Batrachochytrium dendrobatidis* (*Bd*). However, amphibians exhibit different responses to the pathogen, such as survival and population persistence with infection, or mortality of individuals and complete population collapse after pathogen invasion. Multiple factors can affect host pathogen dynamics, yet few studies have provided a temporal view that encompasses both the epizootic phase (i.e. pathogen invasion and host collapse), and the transition to a more stable co-existence (i.e. recovery of infected host populations). In the Sierra Nevada mountains of California, USA, conspecific populations of frogs currently exhibit dramatically different host/ *Bd*-pathogen dynamics. To provide a temporal context by which present day dynamics may be better understood, we use a *Bd* qPCR assay to test 1165 amphibian specimens collected between 1900 and 2005. Our historical analyses reveal a pattern of pathogen invasion and eventual spread across the Sierra Nevada over the last century. Although we found a small number of *Bd*-infections prior to 1970, these showed no sign of spread or increase in infection prevalence over multiple decades. After the late 1970s, when mass die offs were first noted, our data show *Bd* as much more prevalent and more spatially spread out, suggesting epizootic spread. However, across the ~400km^2^ area, we found no evidence of a wave-like pattern, but instead discovered multiple, nearly-simultaneous invasions within regions. We found that *Bd* invaded and spread in the central Sierra Nevada (Yosemite National Park area) about four decades before it invaded and spread in the southern Sierra Nevada (Sequoia and Kings Canyon National Parks area), and suggest that the temporal pattern of pathogen invasion may help explain divergent contemporary host pathogen dynamics.

## Introduction

With thousands of amphibian species experiencing population declines around the world [1], amphibians are facing a global biodiversity crisis, and many suggest this is emblematic of a global mass extinction [2]. Though multiple factors play a role in these declines, the invasion and emergence of the fungal pathogen, *Batrachochytrium dendrobatidis* (*Bd*), and the ensuing epizootics (epidemics in wildlife) are implicated as major contributing factors [3]. *Bd*, and its recently described congener *Batrachochytrium salamandrivorans* (*Bsal*), are the only chytridiomycete known to be pathogenic to vertebrates. Since the description of *Bd*, [4], it has been detected on every continent except Antarctica and is frequently associated with amphibian die-offs [5–8], but not all species are susceptible. *Bd* infects the skin of amphibians and induces a thickening of the skin (hyperkeratosis) on the host, disrupting osmotic balance which, in highly infected individuals, often results in death [4, 9–11].

Two decades after its discovery, the dynamics and emergence of *Bd* are yet to be fully understood. Genomic studies have revealed that there are multiple lineages of *Bd*. For example, the Global Panzootic Lineage (*Bd-*GPL) is associated with *Bd*-epizootics and host population collapse [12], but other lineages are found in areas where epizootics have not been found (e.g. South Korea) [12–15] and many species survive infections. Asia is a geographic hotspot for *Bd* genetic diversity and is proposed as a possible source of the *Bd*-panzootic that began in the 20^th^ century [16]. In the Americas (North, Central and South America), many of the reported declines of amphibians are attributed to *Bd*-GPL epizootics, yet most occurred decades before *Bd* was discovered [17, 18]. Thus, retrospective studies are needed to help create a timeline for *Bd* emergence and spread.

Studies of *Bd* epizootics in California were some of the first to describe host pathogen dynamics of chytridiomycosis in detail [7, 11, 19], yet, like many other areas that have suffered epizootics, the historical view of *Bd* in the region has not been fully described. The earliest evidence of *Bd*-infection in California is from an American bullfrog (*Rana catesbeiana*) specimen collected in 1928 [20]. Although this widely introduced species is identified as a reservoir species for *Bd*, and thus may have facilitated *Bd* invasion [21], there is no evidence that this infection case resulted in epizootics and may represent a failed invasion. Evidence suggests that the timing of *Bd* emergence (i.e. increase in infection prevalence and geographic spread) in California is from the late 1960s to the 1980s [22–24], a time period that coincides with declines of many species in the region [25–27].

In the Sierra Nevada mountain range of California, population declines and local extinctions have been documented in most of the amphibians that occur there [27]. Some population declines are attributed to introduced species like non-native fishes [28], though the causes of other declines are unknown [26]. Between 1976 and 1979, a mass die-off of Yosemite toads (*Anaxyrus canorus*) was documented near Yosemite National Park [29]. A later study suggested it could have been caused by a *Bd* epizootic, but the study was ultimately inconclusive [30]. Bradford (1991) documented a mass mortality event of the southern mountain yellow-legged frog (*Rana muscosa*) in Kings Canyon National Park in 1979; the cause of morality was not determined, but by 1989 the species was extirpated from that area of the park [25].

The southern mountain yellow-legged frog (*Rana muscosa*) and Sierra Nevada mountain yellow-legged frog (*Rana sierrae*) have undergone extensive population declines and local extinctions over the past 100 years [31]. Recently, population collapse and extinction in both species of frog have been shown to be primarily caused by *Bd-*epizootics [6, 7]; however, the extent of the effect across the entire Sierra Nevada range is unknown. For example, studies of host pathogen dynamics in both species of frog have shown that some host populations co-exist with the pathogen [19], while others go extinct < 1 year after pathogen invasion [7]. In the Yosemite area (central Sierra Nevada), mass die-offs associated with *Bd*-epizootics have not been observed in either *R*. *sierrae* or *R*. *muscosa*, yet populations are all infected with *Bd*. These frog populations exhibit moderately high *Bd* infection prevalence (60–75%) and very low *Bd* infection intensity (< 1 Zswab (zoospore equivalents of *Bd* DNA per swab) [19]). However, in the Sequoia-Kings Canyon area (southern Sierra Nevada), *Bd*- epizootics and die offs have been documented in those same species [7]. Here *Bd* infection prevalence rises rapidly to 100% soon after invasion and establishment of *Bd*. During this time, the *Bd* infection intensity rises 3–4 orders of magnitude higher than in Yosemite) (> 10,000 Zswab) [7], at which point, populations collapse. We hypothesize that *Bd* invaded and spread throughout the central Sierra Nevada (Yosemite area) long before it invaded and spread in the southern Sierra Nevada (Sequoia-Kings Canyon area) and propose that this may explain differences in present day host-pathogen dynamics. To test this, we conducted a retrospective survey using museum specimens to describe *Bd*-host dynamics in the Sierra Nevada mountain range over the past century. Because there are fewer available museum specimens from contemporary populations, we also present portions of previously published data on contemporary populations for comparison. Previous studies have hypothesized that *Bd* is an invasive pathogen in California, thus we also investigate the relationship between *Bd* infection and anthropogenic and abiotic factors (i.e., climate variables) that could help explain why Bd became established and spread in some areas. We also use statistical techniques to estimate when *Bd* invasion occurred in the Sierra Nevada, based on our available data dating back over a century.

## Methods

### Sampling of museum specimens

We collected skin swabs from 1165 formalin-fixed, ethanol-preserved, post-metamorphic anurans in museum archives from 1900 to 2005. Using the VertNet.org database, we identified and sampled specimens from permanent collections housed at the California Academy of Sciences, Museum of Vertebrate Zoology, Natural History Museum of Los Angeles County, the Slater Museum of Natural History, and the Carnegie Museum of Natural History. To maximize the probability of detecting *Bd*, we selected all available *R*. *muscosa* and *R*. *sierrae* museum specimens, because these species are known to have undergone dramatic *Bd*-related population declines [7]. In decades where there were <100 *R*. *muscosa* and *R*. *sierrae* specimens available, we randomly sampled other sympatric anuran species (*Anaxyrus canorus*, *Anaxyrus boreas*, and *Hyliola regilla*) within the Sierra Nevada range until we reached the 100-sample size for those decades. All of the data we collected and analyzed are freely accessible on the AmphibiaWeb amphibian disease portal online database (*Butler*, *H*. *2017 "Sierra Nevada Retrospective Analysis" AmphibiaWeb*: *Amphibian Disease Portal <**https*:*//n2t*.*net/ark*:*/21547/Ars2**>*).

We followed the museum swabbing technique described in Cheng et al. (2011) [32]. Each specimen was swabbed a total of 30 strokes with a sterile rayon-tipped swabs (MW113, Medical Wire and Equipment, Corsham, UK): 10 strokes on each side the ventral surface (running from the abdomen towards the pelvis and inner thighs), and 5 strokes on the toes and webbing of each hind foot. To reduce the possibility of cross contamination from multiple specimens kept in the same jar, each specimen was rinsed with 70% ethanol prior to swabbing, held in a unique plastic bag to prevent glove contamination, and gloves were changed between specimens. Swabs were stored in 1.5 mL microcentrifuge tubes and refrigerated at 4°C until extraction. Prior to extraction, swab vials were placed in a SpinVac (Savant Instruments, Farmingdale, NY, USA) for 15–20 min to evaporate any ethanol which could inhibit PCR. DNA extraction from the swabs was done using 40μL of Prepman Ultra (Applied Biosystems, Carlsbad, CA, USA) and diluted 1:10 with 0.25×TE Buffer. Presence of *Bd* was assayed by real-time qPCR, following the method described in Boyle et al. (2004) [33]. Samples were run in duplicate along with negative controls (H_2_0, TE Buffer) and positive controls at dilutions of 100, 10, 1, and 0.1 Zoospore Equivalents. Raw qPCR output was multiplied by a factor of 80 to account for the dilution factor (1:80), giving a relative measure in terms of zoospore equivalents (Zswab) on the specimen. A sample was considered *Bd* positive if the amplification curve was sigmoidal with a Zswab value greater than zero.

Specimen collection localities were plotted in Quantum GIS 3.0 Girona (www.qgis.org). Because not all museum databases or collection labels have been updated to reflect the 2007 taxonomic split of *R*. *muscosa* (*sensu lato*) into allotypic northern *R*. *sierrae* and a southern *R*. *muscosa* (*sensu stricto*) [31], all specimens of “*Rana muscosa”* were checked against range information and were assigned to a species given morphology (*i*.*e*. *R*. *muscosa* having a longer leg length to body size ration compared to *R*. *sierrae*) and locality data [31].

### Statistical analyses

All statistical analyses were performed using the statistical software R (version 3.5.0). For each decade we calculated *Bd* prevalence with 95% binomial confidence intervals (CI). We also calculated the probability of not detecting *Bd* in each decade based on our sample size, assuming a binomial distribution.

We performed a stepwise binomial logistic regression using *Bd* infection status as the response (dependent) variable on the historical survey data, as individuals are either infected (Zswab > 0) or not infected (Zswab = 0). The data were analyzed in two categories: 1) all decades and 2) only the decades before emergence, which we defined as the decades prior to the decade with the greatest change in *Bd* prevalence. We did not perform a regression for post-emergence decades since this study was focused on understanding factors associated with the *Bd* invasion. We used the following explanatory variables in our model: annual mean temperature, annual minimum temperature, annual maximum temperature, elevation, annual precipitation, human footprint, distance to the closest water body, category of the closest water body, croplands, built environment, population density, roads, railways, and pastures. Using the scale function in R’s base package, we scaled the covariates using their mean and standard deviation to enable a better comparison of coefficients [34]. Elevation and topographic information for the distance to the closest water body were downloaded from the US Geological Survey National Hydrography Dataset (nationalmap.gov, https://nhd.usgs.gov/data.html) using the package FedData (version 2.4.6) and distance to the closest water body was calculated using the gdistance package (version 1.2–1). Annual temperature and precipitation information were downloaded from PRISM (PRISM Climate Group, Oregon State University, http://prism.oregonstate.edu, created 10 Feb 2018), and human footprint and land use information were accessed through the Dryad digital repository listed in Venter et al. (2016) [35], and the data extracted using the R package raster (2.5–8). We performed a Pearson correlation test to determine if any of the explanatory variables were highly correlated with each other (r > 0.9 or r < -0.9) and eliminated the variables that were highly correlated to reduce multicollinearity. The eliminated highly correlated variables were minimum temperature, maximum temperature, and elevation–all of which are highly correlated with mean temperature. We validated the models using *k*-fold cross validation.

We conducted Bayesian hierarchical modeling using Markov Chain Monte Carlo (MCMC) to estimate the arrival year of *Bd* in the Sierra Nevada with the R package rjags (version 4–6). In this model, *Bd* arrival is described using a threshold model where *Bd* switches from absent to present in the population with some mean prevalence. The number of infected individuals in each year was treated as a draw from a binomial distribution with a sample size equal to the number of individuals sampled that year [36, 37].

As a baseline for *Bd* infection prevalence comparison, we used a conservative probability of 0.05. This probability is based on a previous study that used the same qPCR technique on museum specimens and showed a mean of 11% infection prevalence in a population where *Bd* was determined to be endemic (Illinois, USA) for specimens collected over a 100-year timespan [38].

## Results

### Museum sampling

**Fig 1:**
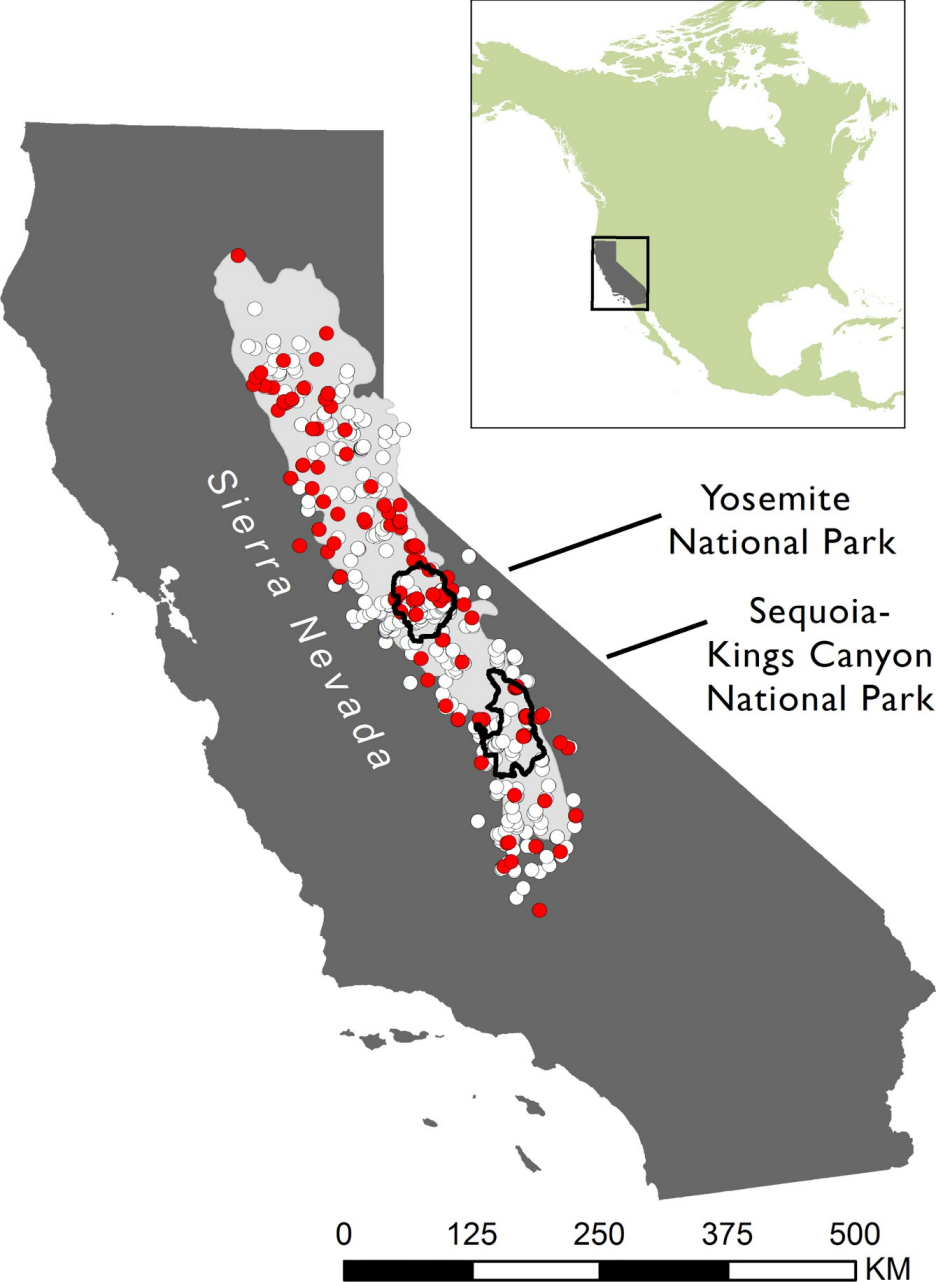
Spatial distribution of 1165 amphibian museum specimens tested for *Bd-*infection collected between 1900–2005 in the Sierra Nevada. Red and gray circles denote individuals tested positive and negative for *Bd*; respectively.

**Fig 2:**
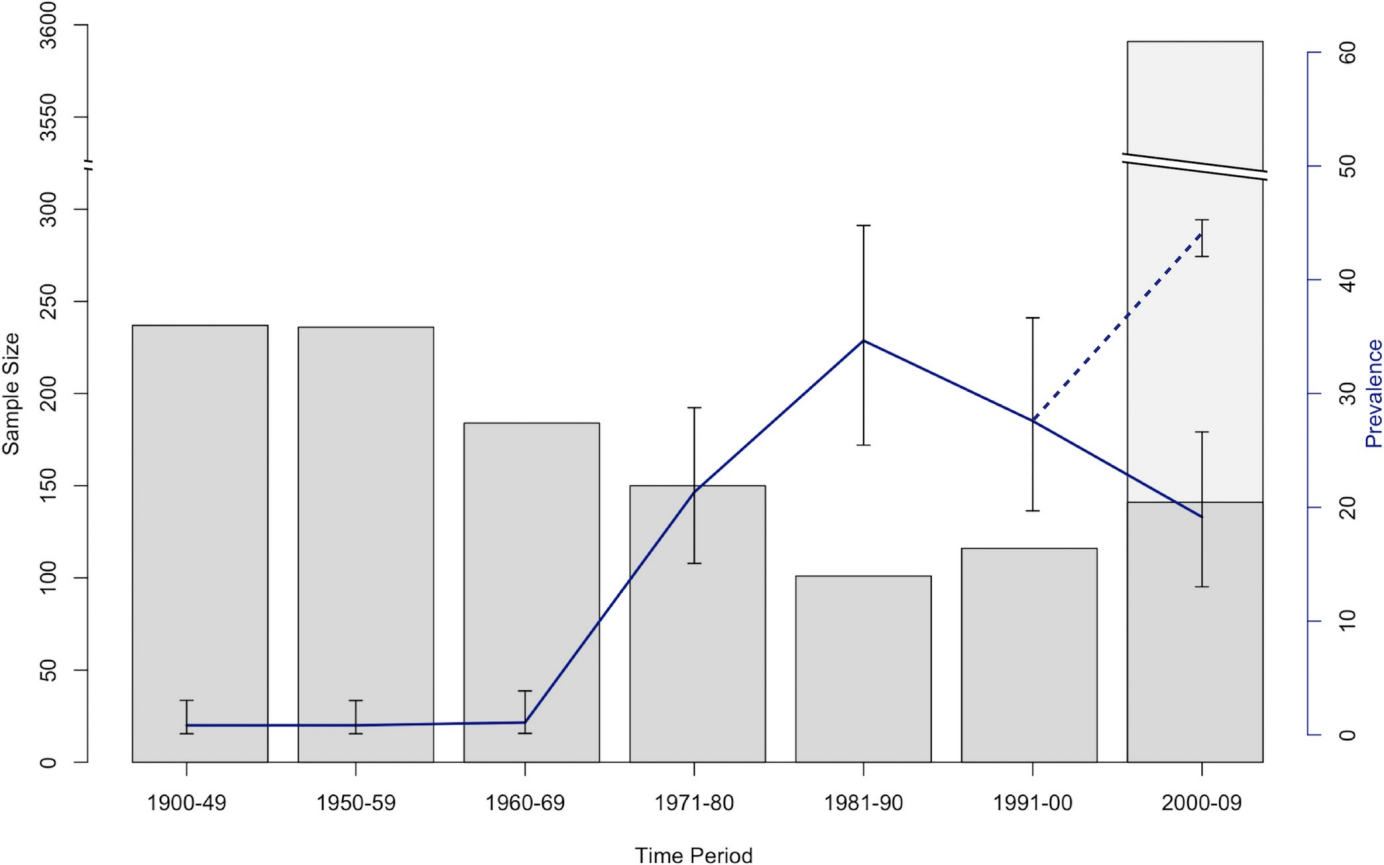
*Bd* infection prevalence in anurans of the Sierra Nevada from 1900–2009. Bar graphs denote sample size from each time period. Dark gray bars denote samples from museum specimens, and light gray bars denote samples collected from live animals in the field (live animal data from [7]). Blue line denotes *Bd* infection prevalence calculated from museum specimens only, and dotted blue line denotes *Bd* infection prevalence including both museum specimens and live animals in the field (i.e. museum specimens and data from [7]).

**Fig 3:**
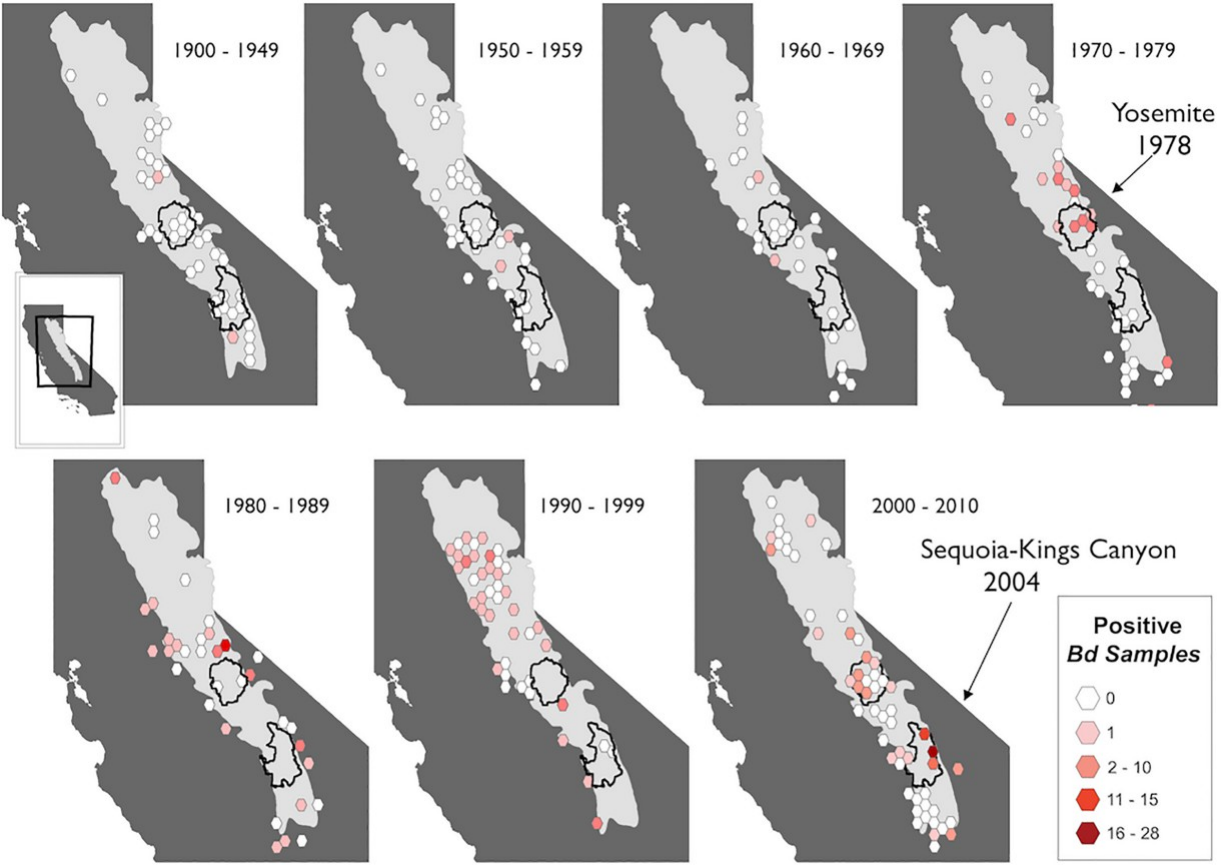
New incidences of *Bd*-positive amphibians in the Sierra Nevada mountain range per time period in California from 1900–2009. The earliest *Bd* positives (12/26) were detected in Yosemite National Park were in the 1970s (mass die offs documented there in 1978 [Sherman and Morton 1993]). The first *Bd* positives (1586/3492) detected in Sequoia- Kings Canyon National Parks were in the 2000s (mass die offs documented there beginning in 2004; [7]). The Sierra Nevada mountains are denoted by light gray shading.

A total of 132 out of 1164 archived specimens sampled across the Sierra Nevada were *Bd*-positive (Fig 1); six were collected before 1970 and the remaining 126 positives were collected after 1970 (Table 1; Fig 2). The probability of not detecting *Bd*, given our sample size, was low (p < 0.01) for each time period (Table 1). The pre-1970 *Bd*-positive specimens were collected in 1939, 1942, 1955, 1959, 1962, and 1965, at isolated and widely distributed sites across the mountain range (Fig 3). The overall *Bd* infection prevalence by decade ranged from 0.8% in the 1950s to 34.7% in the 1980s, with the greatest change occurring in the 1970s (Fig 2). When we included previously published data collected from live animals in the field (n = 3492) [7], the prevalence in the 2000s was over 40% (Fig 2). Based on the museum data, the Bayesian hierarchical modeling iterations predicted that *Bd* would have likely arrived between 1932–1939 (95% of iterations; $\bar{x}$ = 1936) in the Sierra Nevada, though this arrival time may also signal a failed invasion attempt.

**Table 1:** Sample size per decade and the probability of detecting no *Bd* based on a 5% conservative probability of *Bd* detection. Credible intervals (CI) were calculated using binomial confidence intervals.

Time Period	Negatives	Positives	n	Lower CI	Upper CI	Pr
1900–1949	235	2	237	0.1	3.01	< 0.01
1950–1959	234	2	236	0.1	3.03	< 0.01
1960–1969	182	2	184	0.13	3.87	< 0.01
1970–1979	118	32	150	15.07	28.76	< 0.01
1980–1989	66	35	101	25.46	44.77	< 0.01
1990–1999	84	32	116	19.69	36.65	< 0.01
2000–2009	114	27	141	13.01	26.62	< 0.01

**Fig 4:**
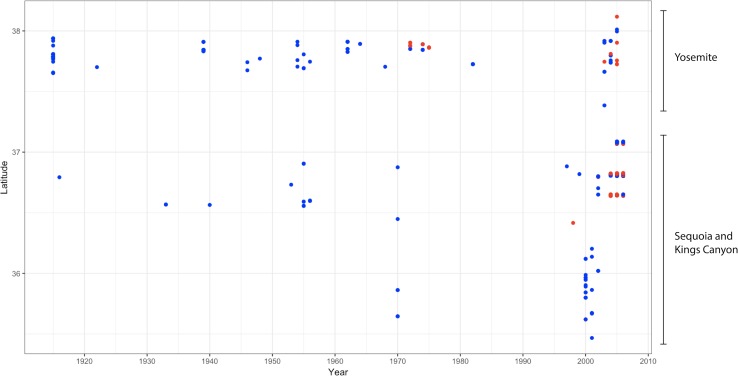
Chronology of *Bd* infected amphibians collected in and around Yosemite National Park (central Sierra Nevada) and Sequoia-Kings Canyon National Parks (southern Sierra Nevada).

We found that *Bd*-positive specimens appeared earlier and in higher numbers in the central and northern part of the Sierra Nevada range (*e*.*g*. the vicinity of Yosemite National Park and northwards), compared to the southern areas (*e*.*g*. the vicinity of Sequoia-Kings Canyon National Parks; Fig 3), but few samples were collected in the southern areas between the late 1970s and 1990s. In the Yosemite National Park area, the first positive was detected in 1972, and the first mass declines noted in 1978. In the Sequoia-Kings National Parks area the first detection of *Bd* was in 1998, with the first evidence of a rapid increase in *Bd* prevalence being in 2004 (Fig 4). We also detected several early positives from animals collected in 1976 in southwest Inyo County in the most extreme southern reaches of the Sierra Nevada, far south of the Sequoia-Kings National Parks area (Fig 3).

The best model for the stepwise binomial logistic regressions, using *Bd* presence as a response variable, (Table 2) shows differences in which scaled coefficients were significant at predicting *Bd-*positive individuals between pre-1970’s and all time periods. The best model for all decades shows both human factors (human footprint, croplands, built environment, human population density, roads, and railways), and climatic variables (precipitation and mean temperature) as significant predictors of *Bd* infections (Table 3). For the pre-1970s time period (before *Bd* emergence), anthropogenic factors (built environment, human footprint, and railways) are significant predictors of *Bd* infection (Table 4). Models that included amphibian species as a factor did not suggest that that factor was significant in the context of this study. Our *k*-fold cross validation for all time periods and pre-1970s models show a cross-validation estimation of accuracy of 88.7% and 75.5%; respectively.

**Table 2:** Lowest AIC models for stepwise binomial logistic regression.

	All Time Periods		Pre-1970s	
*Predictors*	*Odds Ratios*	*p*	*Odds Ratios*	*p*
(Intercept)	0.11	**<0.001**	0.31	**<0.001**
ppt	1.63	**<0.001**	1.15	0.111
tmean	1.57	**<0.001**		
HFP 2009	5.57	**0.001**	3.43	**0.003**
croplands 2005	0.81	0.086	0.82	0.134
Built 2009	0.26	**<0.001**	0.37	**0.011**
Popdensity 2010	0.62	**0.009**		
Roads	0.48	**0.016**	0.66	0.087
Railways	0.71	**0.034**	0.76	**0.041**
distwater			1.19	0.108
Pasture 2009			0.84	0.135
AIC	783.788		564.465	

**Table 3:** Selection for the lowest AIC model for all time periods sampled.

Predictors	Model 1	Model 2	Model 3	Model 4
Precipitation	X	X	X	X
Mean Temperature	X	X	X	X
Human Footprint Index	X	X	X	X
Croplands	X	X	X	X
Built Environment	X	X	X	X
Population Density	X	X	X	X
Roads	X	X	X	X
Railways	X	X	X	X
Category of Closest Water Body				X
Distance to Closest Water Body		X	X	X
Pasturelands			X	X
AIC	783.79	785.02	786.70	792.79
Resid. Dev	765.79	765.02	764.70	756.79
Resid. Df	1155.00	1154.00	1153.00	1146.00
Deviance	0.77	0.31	7.91	

**Table 4:** Selection for the lowest AIC model for pre-1970s.

Predictors	Model 1	Model 2	Model 3	Model 4
Precipitation	X	X	X	X
Mean Temperature			X	X
Human Footprint Index	X	X	X	X
Croplands	X	X	X	X
Built Environment	X	X	X	X
Population Density	4	X	X	X
Roads	X	X	X	X
Railways	X	X	X	X
Category of Closest Water Body				X
Distance to Closest Water Body	X	X	X	X
Pasturelands	X	X	X	X
AIC	564.47	566.08	568.06	575.02
Resid. Dev	546.47	546.08	546.06	541.02
Resid. Df	498.00	497.00	496.00	490.00
Deviance	0.39	0.02	5.04	

## Discussion

The global collapse of amphibian species caused, in part, by the Bd panzootic, stands as an example that we have entered a sixth mass extinction [2]. Because the pathogen was discovered and described after causing epizootics in Central America and Australia [4, 9], retrospective studies are vital to improve our understanding of *Bd*’s pathogen invasion and spread. Despite sampling biases of museum specimens that were collected for reasons unrelated to disease ecology, retrospective studies, like this one, can provide insight regarding pathogen invasion history and disease dynamics.

Our survey of museum specimens collected over a 100-year period in the Sierra Nevada found no evidence of *Bd* before 1939. For almost the next four decades, we detected only a few *Bd*-infected frogs and no sign of spread or an increase in infection prevalence until the late 1970’s. These results are consistent with a growing body of evidence showing that *Bd* may have invaded and spread in California and the west coast of North America approximately a decade before mass die offs were discovered [21, 22, 23, 36]. Unfortunately, those studies did not provide disease data from the Sierra Nevada, where studies first described pathogen host dynamics during epizootics that resulted in local host extinctions [7, 19]. However, one previous retrospective study did discover a *Bd*-positive Yosemite toad (*Anaxyrus canorus*) collected at Tioga Pass Meadow in 1978, the year before a mass population die-off at the site (Green & Sherman 2001). We also detected *Bd-*infected *R*. *sierrae* in the vicinity of Tioga Pass before the population collapse, suggesting that a *Bd* epizootic may have swept through the Yosemite area in the mid to late 1970s.

We found evidence of *Bd* invasion and spread that varies geographically and temporally across the Sierra Nevada mountain range. Unlike previous historical studies that occurred over very large (continental) or limited (1–2 Km) geographic scales [5, 7], we did not detect a directional wave-like spread of *Bd* across the entire mountain range. Instead, we found *Bd* invaded host populations asynchronously in separate regions across the 400 km^2^ area, but specimens were collected for purposes not related to this study, and this limited our ability to detect a wave. In the central Sierra Nevada (Yosemite area), *Bd* was first detected in the 1970s (Fig 3), and epizootics are suggested to have occurred soon thereafter (Sherman and Morton 1993). In the southern Sierra Nevada (Sequoia-Kings Canyon area), *Bd* invaded more recently in the 2000s (Fig 3), and epizootics have been documented in 2004–2008 [7]. Interestingly, these areas currently have different *Bd*-host dynamics, with frog populations in the central Sierra Nevada persisting in an enzootic state with *Bd* [19, 39] while frog populations in the southern Sierra Nevada are now experiencing epizootics and collapse [7]. Although the invasion patterns could be an artifact of the biased spatial-temporal spread of the museum specimens, our results indicate that *Bd* may have invaded these separate geographical areas at different times, and that difference in timing could account for the varying present-day *Bd*-host dynamics at these locations.

There could be other explanations for the differing present-day dynamics of these frog populations. It could be a result of differences in the pathogen, differences in the host or host community, and/or differences in habitat (abiotic factors). The virulence hypothesis states that pathogens gradually lose their highly virulent invasive qualities in order to maintain themselves in host populations [40, 41]. *Bd* may have undergone selection for lower virulence, or the host populations might have undergone selection for higher resistance to *Bd*. Host genotype, such as MHC II, have been associated with *Bd*-resistance [42, 43]. The arrival of a known *Bd* reservoir, such as American bullfrogs (*L*. *catesbeiana*) into hosts populations could also explain the contrasting dynamics [21], along with changes in temperature which could limit *Bd* growth [44]. However, a recent study in the Sierra Nevada found that neither differences in *Bd* strains from Yosemite and Sequoia-Kings Canyon nor abiotic factors explained differences in host susceptibility [39]. Instead, differences in host-pathogen dynamics were explained by the geographic location of the frog populations; frogs from the Yosemite area were less susceptible to *Bd* than frogs from the Sequoia-Kings area [39]. This further supports the hypothesis that the timing of pathogen invasion may explain present day differences in *Bd* host dynamics in the contemporary populations in these areas.

Infected frogs found prior to the 1970s may represent failed invasions, a pathogen invasion that took decades to establish, or may be evidence of the presence of non-virulent, endemic lineages of *Bd*. Currently, the only known lineage of *Bd* in the Sierra Nevada is the Global Panzootic Lineage (*Bd-*GPL) [12,14], but in other areas (*e*.*g*. Brazil) virulent and non-virulent lineages of *Bd* have been found in the same populations of hosts [45]. More studies are needed to determine which *Bd* lineages are present in the Sierra Nevada.

At the larger spatial scale of the Sierra Nevada range, our regression analyses show that anthropogenic factors likely played a role in the arrival and spread of *Bd*, which is similar to previous studies in California [21–24]. We found that, human footprint index in general, and railways, and built environment specifically, were significant predictors of frog specimen being a *Bd* positive (Table 2). These results support the idea that *Bd* invaded the high country (areas above 2500m) of the Sierra Nevada earliest in areas with the most human influence, such as Tioga Pass road in Yosemite National Park. The last area of the Sierra Nevada to become infected by *Bd*, almost four decades later, appears to be the Sequoia-Kings Canyon National Parks area, where the largest wilderness areas are located and access by humans is limited.

Archived amphibian museum specimens provide important historical insights that help increase our understanding of the host-pathogen dynamics of *Bd* at a local, regional, and global scale. For example, a study in Central America showed *Bd* invasion coincident with collapse of amphibian communities [32], and this helped explain the timing and losses of amphibian species that had been proposed as *Bd*-epizootics [5]. Other studies have found a 100-year history of amphibians co-occurring with *Bd* and no evidence of *Bd* invasion in areas with no known history of *Bd* epizootics [38, 46]. Here we show an increase in *Bd* prevalence before collapse and disappearance of many amphibians in two geographically separate locations within the Sierra Nevada four decades apart. We found that *Bd* became established and began to increase in prevalence in the late 1970’s in the central Sierra Nevada (Yosemite area), which is coincident with declines in those areas [26, 29], and is consistent with the hypothesis that chytridiomycosis was associated with declines recorded in several Sierra Nevada amphibian species [2, 26, 29]. We also found *Bd* invasion prior to documented epizootics in the 2000s in the southern Sierra Nevada [7]. Last, we detected early presence (before the 1970s) of *Bd* at low prevalence in spatially spread out locations that apparently did not lead to epizootics, which may be indicative of either an endemic but less virulent lineage of *Bd* or failed historic invasions. Further retrospective studies in conjunction with current field and lab studies are needed to improve our understanding of pathogen invasion history and host-pathogen dynamics.

## Supporting information

S1 Table The number of specimens of each host species sampled by time period. (PDF)
