# Supplementary material for: Pathogen invasion history elucidates contemporary host pathogen dynamics
Source: PLoS One. 2019 Sep 19;14(9):e0219981. doi: 10.1371/journal.pone.0219981 (PMC6752790; doi:10.1371/journal.pone.0219981)
Supplement: S1 Table — (PDF) [file pone.0219981.s001.pdf]

Species	1900	1910	1920	1930	1940	1950	1960	1970	1980	1990	2000	Total
Bufo boreas						1	2	17	12	6	13	51
Bufo sp.								7				7
Bufo canorus									1		1	2
Pseudacris regilla						35	44	31	60	61	102	333
Rana boylii		10				55	11	14		8	4	102
Rana cascadae								1				1
Rana catesbeiana						1	1	2	5	25	14	48
Rana draytonii							1					1
Rana muscosa		51	1	11	5	26	58	8		15	2	177
Rana pipiens							4					4
Rana sierrae	1	61	11	58	28	118	63	70	23		5	438
**Total**	1	122	12	69	33	236	184	150	101	115	141	1164

Table S1. The number of specimens of each host species sampled by time period
